# Screening food-borne and zoonotic pathogens associated with livestock practices in the Sumapaz region, Cundinamarca, Colombia

**DOI:** 10.1007/s11250-017-1251-6

**Published:** 2017-03-11

**Authors:** Nelson E. Arenas, Diego A. Abril, Paola Valencia, Surabhi Khandige, Carlos Yesid Soto, Vilma Moreno-Melo

**Affiliations:** 1 Faculty of Agricultural Sciences, Universidad de Cundinamarca, Diagonal 18 No. 20-29, Fusagasugá, Cundinamarca 252211 Colombia; 2 Department of Biochemistry and Molecular Biology, University of Southern Denmark, Campusvej 55, 5230 Odense M, Denmark; 3 Department of Chemistry, Faculty of Sciences, Universidad Nacional de Colombia, Carrera 30, No. 45-03, Ciudad Universitaria, Bogotá, Colombia

**Keywords:** Bovine brucellosis, Bovine tuberculosis, *Escherichia coli*, *Staphylococcus aureus*, Zoonoses, Antibiotic resistance

## Abstract

Hazardous practices regarding antibiotics misuse, unsanitary milking procedures, and the commercial sales of raw milk and unpasteurized dairy products are currently being practiced by livestock farmers in the Sumapaz region (Colombia). The purpose of this study was to screen for food-borne and zoonotic pathogens associated with local livestock practices. We evaluated 1098 cows from 46 livestock farms in the Sumapaz region that were selected by random. Of the total population of cattle, 962 animals (88%) were tested for bovine TB using a caudal-fold tuberculin test and 546 (50%) for brucellosis by a competitive ELISA. In the population tested, 23 cows were positive for *Brucella* sp. representing a 4.2% seroprevalence and no cases of bovine tuberculosis were found. In addition, food-borne contamination with *Escherichia coli* and *Staphylococcus aureus* was assessed together with antibiotic susceptibility for ten different antibiotics in milk samples from 16 livestock farms. We found that 12 of the farms (75%) were contaminated with these food-borne pathogens. Noteworthy, all of the isolated pathogenic strains were resistant to multiple antibiotics, primarily to oxytetracycline and erythromycin. Our findings suggest that livestock products could be a source of exposure to *Brucella* and multidrug-resistant *E. coli* and *S. aureus* strains as a result of unhygienic livestock practices in the Sumapaz region. Training in good farming practices is the key to improving safety in food production.

## Introduction

Prevailing food regulations demand high-quality assurance and safety in the production chain (Grace 2015). Meeting these global safety standards mandates significant changes in farming practices and animal husbandry (Van Boeckel et al. 2015). The Colombian Agriculture and Livestock Institute (Spanish abbreviation: ICA) is the local authority that assesses compliance with international guidelines, infectious disease control, and compulsory epidemiological registration. The ICA’s programs include the screening and epidemiological surveillance of zoonotic diseases associated with livestock and agricultural farming. These programs are based on good farming practices (GFPs) for animal production food safety which are procedures formulated to contribute to sustainable livestock production in the country (Roess et al. 2015). In fact, GFPs have demonstrated excellent results in farm productivity and have contributed to local development, the maintenance of good animal health and increasing economic profits for farmers (Crowder and Reganold 2015). GFPs have been focused on the control of brucellosis and tuberculosis (TB), which are considered to have the greatest impact among zoonotic diseases in Colombia and South America.

Zoonotic disease eradication includes screening programs and the epidemiological surveillance of food-borne pathogens as key strategic activities (Grace 2015). In the Sumapaz region, livestock farming is the central economic activity; it is performed empirically by local farmers without an in-depth understanding of GFPs. Epidemiological surveillance is important because the commercialization of unpasteurized dairy products can pose a threat to human public health. In addition, zoonotic infections, such as brucellosis and TB, are common, insidious, and persistent problems for livestock farmers in general in developing countries (Aznar et al. 2014; Roess et al. 2015). Despite control measures and sanitation efforts, zoonotic disease outbreaks have been reported in Colombia (Morales and Combariza 2004; González et al. 2007; Tique et al. 2009, 2010). In 2013, brucellosis was the most common zoonotic infection in Colombia; 12,000 cases are reported annually and 343 cases of these occur in the Sumapaz region and neighboring areas. Consequently, a decrease in milk production from 168,216 L/day in 2007 to 117,538 L/day in 2009 (30% decrease) has been reported in the Sumapaz region. In contrast, the bovine TB prevalence has been estimated to be less than 1% in Colombia (De Waard 2010). Along with its economic impact, the morbidity and mortality associated with such zoonotic infections such as brucellosis and bovine TB are also relatively unknown or underestimated by livestock farmers and government institutions. Our purpose was to develop a screening and sanitation program in accordance with GFPs that would involve local farmers in the control of zoonotic and food-borne diseases such as brucellosis and TB to improve animal health.

## Material and methods

### Study area and sampling

**Fig. 1:**
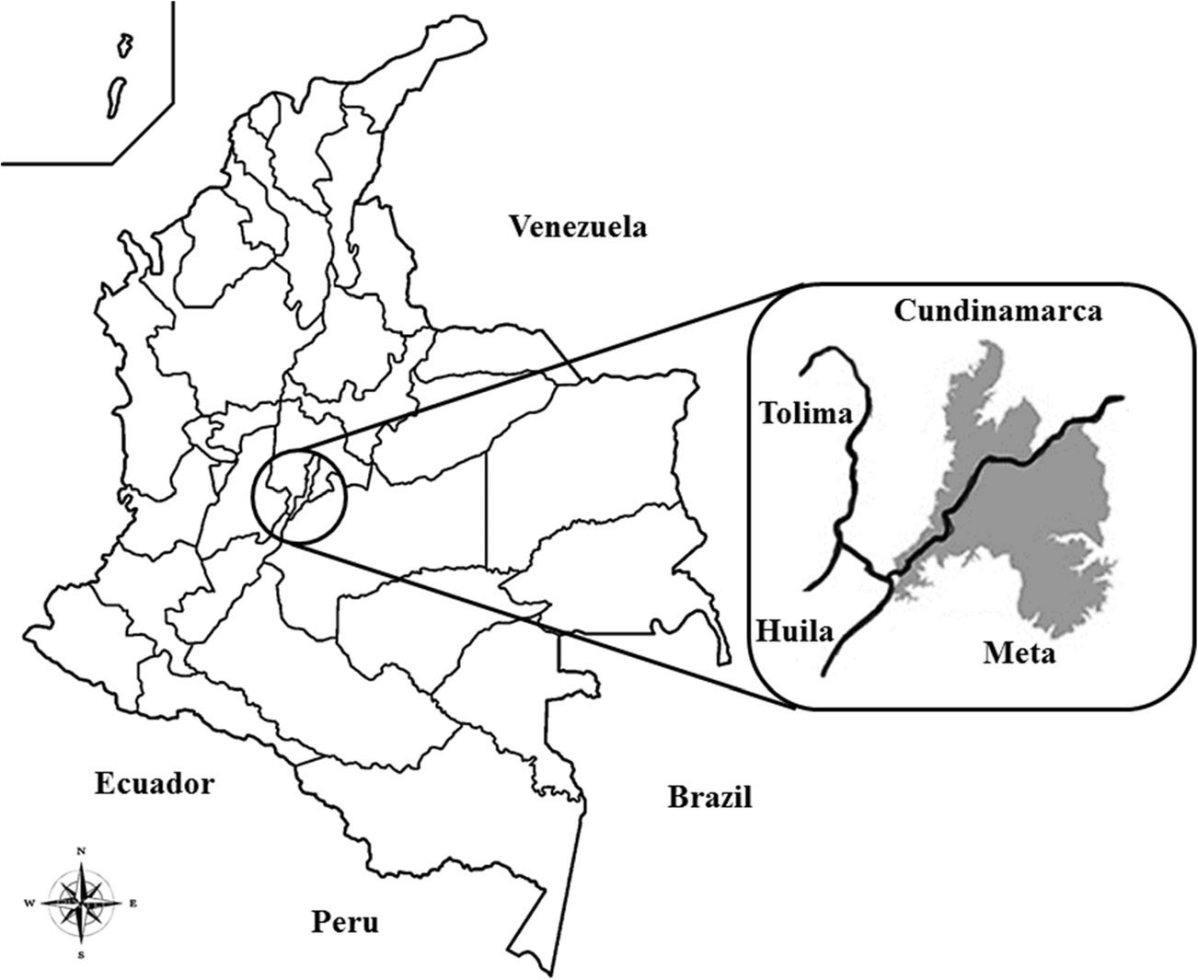
Geographical location of the Sumapaz region in Cundinamarca, Colombia. *Shadow* area inside the *circle* represents the location of the Sumapaz area

A cross-sectional study was performed from January 2014 to June 2015 to detect and identify zoonotic and food-borne pathogens associated with livestock practices in the Sumapaz region. With an area of 1808 km^2^, the Sumapaz region is located in the south of Cundinamarca (representing 8% of the province) and is delimited by Tequendama and Soacha in the north, the province of Tolima in the south, Bogotá in the east, and Alto Magdalena in the west (Fig. 1). Livestock farming and animal husbandry is an important economic activity in this region; thus, 46 livestock farms were randomly selected from Fusagasugá, Silvania, Tibacuy, Arbeláez, Cabrera, Pandi, and Pasca municipalities. Paradoxically, livestock farming is performed empirically without quality assurance schemes and marketing of raw milk and unpasteurized dairy products is currently performed in these municipalities. Five breeds of cattle have been recorded including a local breed (named “Creole”), Holstein, Pardo, Normande, and Cebu (called “Mestizo”). One thousand ninety-eight animals (spread over 46 livestock farms randomly selected in the Sumapaz area) were screened for bovine TB and brucellosis. Of the total population of cattle, 962 animals (88%) were tested for bovine TB and 546 (50%) for brucellosis. Out of 46 livestock farms, 16 were based on milk production. In each of them, ten milk samples were aseptically collected from different random selected cows and were pooled in a bulk tank for further microbiological analyses. In addition, nutrition analysis in raw milk pools was determined using an ultrasonic milk analyzer (LactoScan system, Milkotronic, Ltd., Nova Zagora, Bulgaria) testing: the protein percentage, fat percentage, milk solid nonfat, density, pH, lactose percentage, and total solid percentage.

### Bovine tuberculosis and brucellosis detection

For *Brucella* diagnosis, 10 mL of blood was collected in sterile vacutainer tubes and stored at 4 °C until required. Samples were centrifuged at 3000×*g* for 10 min and processed according to guidelines (ICA 2010). A competitive enzyme-linked immunosorbent assay (ELISA) (Boehringer Ingelheim, Uppsala, Sweden) was performed consistently with the previously standardized technique (Rivera et al. 2003). Results were expressed as percent inhibition by each serum considering a value ≥30% was interpreted as a positive and <30% as negative test. On the other hand, caudal-fold tuberculin tests (CFTs; Thermofisher Scientific, Schlieren-Zurich, Switzerland) were performed by ICA’s certified veterinarians for TB screening. Briefly, the animals were injected intradermally with purified protein derivative (PPD) tuberculin, and the injection site was measured 72 h later. Hypersensitivity reactions, such as swelling or discoloration, were interpreted according to ICA standard procedures (ICA 2004).

### Bacterial identification

For microbiological tests, approximately 15 mL of pooled milk was aseptically collected in sterile tubes and stored at 4 °C. Milk dilutions were streaked in LB plates and incubated at 37 °C to obtain single colonies. Colonies were isolated and classified according to morphological features, shape, and Gram staining. Bacterial identification was performed by using phenotypic methods based on biochemical reactions and bacterial metabolism (sugar fermentation, indole, methyl-red, Voges-Proskauer, motility, gas production, and citrate tests) for Gram-positive and Gram-negative bacteria. Bacterial isolates were tested for antibiotic susceptibility to ten drugs that are commonly prescribed for veterinary use. Antimicrobial susceptibility tests were performed by Kirby-Bauer disk diffusion method in Mueller-Hinton agar plates and interpreted according to the guidelines of the National Committee for Clinical Laboratory Standards (NCCLS) and national regulations for antibiotic use in livestock practices (Ministerio de Salud y Proteccion Social 2013). Eighty percent of the veterinary antibiotics selected (8/10) have broad antibacterial activity against Gram-negative and Gram-positive bacteria involving different drug mechanisms. Data about current livestock practices were assessed with regard to milking routines, tool sterilization, and drug records gathered from livestock smallholders participating in this study.

### Ethical considerations

All cattle tests followed the laws and regulations recommended by the European Communities Council Directive of November 24, 1986 (86/609/EEC) and were in agreement with the ICA’s regulations. Furthermore, in all cases, the livestock farmers were informed verbally about the procedures, risks, and benefits associated with participation in the study.

## Results

To evaluate zoonotic disease control in the livestock farms (*n* = 46), bovine TB and brucellosis were screened through CFTs and competitive ELISA, respectively.

No cases of bovine TB were detected and 23 (4.2%) sera from clinically normal cattle were positive for brucellosis. Nineteen of the brucellosis cases are presented in Table 1. The animals represented were all female and belonged to the following breeds: Brahman (*n* = 9), Cross-bred (*n* = 3), Bon (*n* = 3), Holstein (*n* = 1), Normande (*n* = 1), Creole (*n* = 1), and unknown (*n* = 1). All sera showed inhibition levels between 47 and 96% in the competitive ELISA tests. Furthermore, 30 livestock farmers who currently performed milking routines on the farms with index cases (brucellosis) were also tested, but were found to be negative for brucellosis and bovine TB.

**Table 1:** Characteristics of brucellosis infection in cattle. Inhibition ≥30% indicated a positive test, and inhibition <30% indicated a negative test. The results were obtained with competitive ELISA

Location	Municipality	Breed	% inhibition	Age (months)
1	Arbeláez	Brahman	54	25
2	Arbeláez	Brahman	64	27
3	Arbeláez	Brahman	47	45
4	Arbeláez	Brahman	47	43
5	Arbeláez	Brahman	60	45
6	Arbeláez	Brahman	48	41
7	Arbeláez	Brahman	50	29
8	Arbeláez	Brahman	48	24
9	Arbeláez	Brahman	56	47
10	Arbeláez	Cross-breed	89	64
11	Arbeláez	nd	>30	nd
12	Fusagasugá	Cross-breed	96	104
13	Fusagasugá	Cross-breed	96	30
14	Fusagasugá	Bon Cebu	75	37
15	Fusagasugá	Bon	47	58
16	Fusagasugá	Bon Cebu	50	30
17	Pasca	Holstein	96	55
18	Pasca	Normand	>30	nd
19	Pandi	Creole	53	72

*nd* data non-determined

**Fig. 2:**
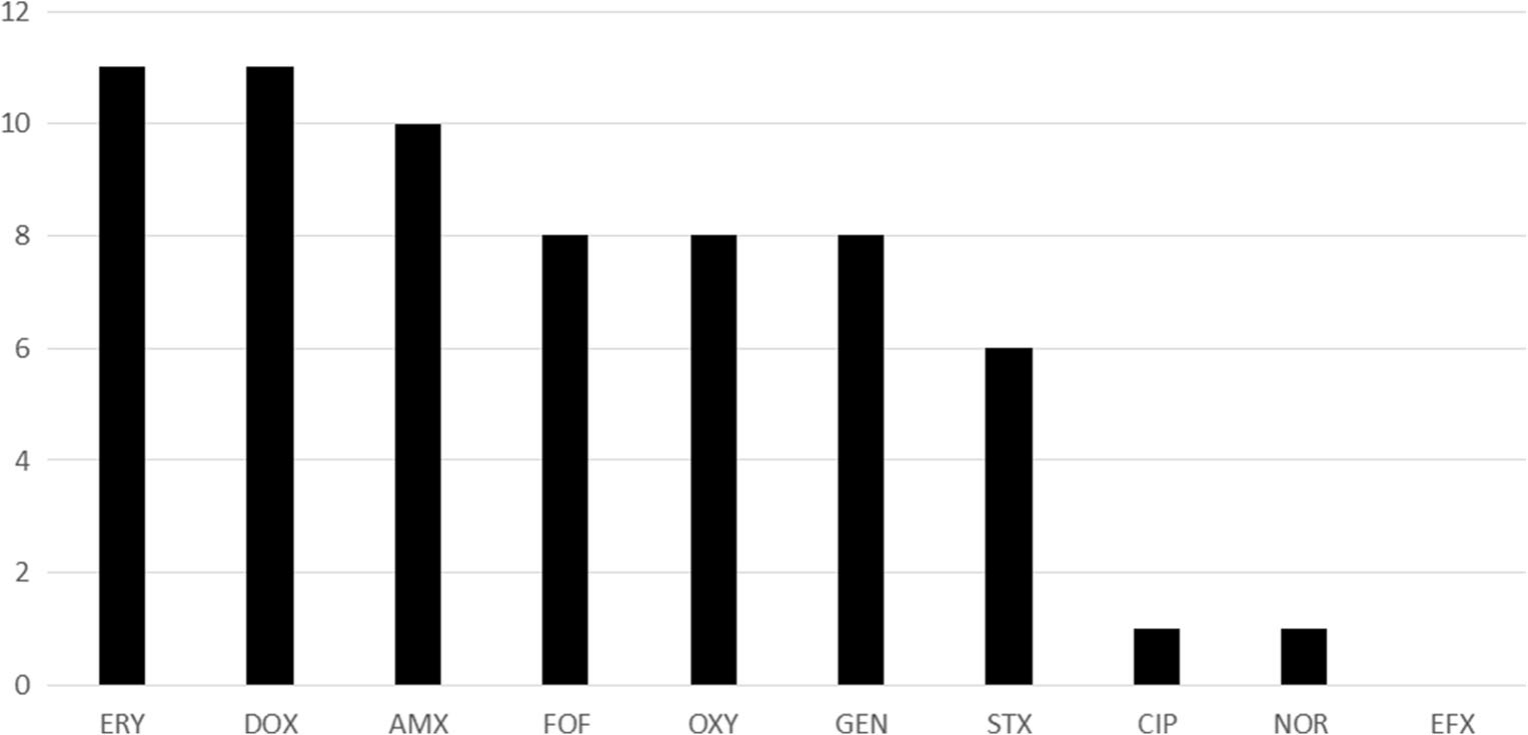
Frequency of antibiotic resistance of each drug tested in *E. coli* and *S. aureus* isolates. *FOF* fosfomycin, *SXT* trimethoprim-sulfamethoxazole, *AMX* amoxicillin, *CIP* ciprofloxacin, *OXY* oxytetracycline, *NOR* norfloxacin, *EFX* enrofloxacin, *GEN* gentamicin, *ERY* erythromycin, *DOX* doxycycline

To assess milk quality and safety before GFP achievement, nutritional composition and microbiological tests were performed in the 16 milk pooled samples. Nutrition analysis did not show any significant differences in nutritional content between samples (data not shown). Nevertheless, the majority of milk pooled samples showed microbial contamination. Thus, cultures of four pooled samples were negative, while 12 of the milk samples (75%) indicated bacterial contamination with *Staphylococcus aureus* (*n* = 1) and *Escherichia coli* (*n* = 11). None of the bacterial isolates displayed sensitivity to any of the tested antibiotics (other than enrofloxacin), instead most of them showed extended drug resistance. All *E. coli* isolates presented multiple resistance to at least four different antibiotics in 90% (*n* = 11) of all cases. The most concerning findings were that four of the examined *E. coli* isolates (33.3%) were resistant to seven of the tested antibiotics, while two other *E. coli* isolates (16.7%) showed resistance to five to six antimicrobial drugs (Table 2). A single *S. aureus* isolate was found to be resistant to oxytetracycline (OXY) and erythromycin (ERY). Antibiotic resistance involving DOX and/or ERY was often detected in 11 (92%) strains; ten strains showed amoxicillin (AMX) resistance (83%) and eight of the bacterial isolates (67%) showed resistance to fosfomycin (FOF), OXY, and gentamicin (GEN) (Fig. 2). Overall, this study reports a very high prevalence of multidrug-resistant strains of *E. coli* involving livestock farming in the Sumapaz region. The milk contamination observed in the course of our study could be explained by the fact that 91% of livestock farmers performed milking routines manually without gloves and missing knowledge about possible sources of bacterial contamination. These practices did not include the sterilization and cleaning of milking tools, and 55% of the farmers included in our study lacked proper records regarding the administration of drugs and medicines to their livestock herds.

**Table 2:** Detection of multidrug antibiotic resistance in pathogens (*E. coli* and *S. aureus*) associated with livestock farming in the Sumapaz region. The thresholds for minimum inhibitory concentrations were assessed using local regulations for livestock farming

Number of isolates (%)	Number of antibiotics showing resistance	Antimicrobial resistance pattern (number of isolates)
1 (8.3)	2	OXY, ERY (1)
3 (25)	4	AMX, GEN, ERY, DOX (1)
		FOF, AMX, ERY, DOX (1)
		FOF, SXT, NOR, DOX (1)
2 (16.7)	5	SXT, AMX, GEN, ERY, DOX (1)
		FOF, AMX, OXY, ERY, DOX (1)
2 (16.7)	6	FOF, AMX, OXY, GEN, ERY, DOX (2)
4 (33.3)	7	SXT, AMX, CIP, OXY, GEN, ERY, DOX (1)
		FOF, SXT, AMX, OXY, GEN, ERY, DOX (3)

*FOF* fosfomycin, *SXT* trimethoprim-sulfamethoxazole, *AMX* amoxicillin, *CIP* ciprofloxacin, *OXY* oxytetracycline, *NOR* norfloxacin, *EFX* enrofloxacin, *GEN* gentamicin, *ERY* erythromycin, *DOX* doxycycline

## Discussion

The adoption of GFP guidelines represents an important step in the development of sustainable agriculture in the Sumapaz region (Cundinamarca, Colombia). Many livestock farmers have a low income and lack required knowledge about GFPs, which leads to low milk productivity and poor quality together with commercial value loss. Therefore, GFP training is essential for helping farmers achieve high-quality standards and develop sustainable livestock practices (Leibler et al. 2016; Abbasi and Abbasi 2016). In Colombia, antibiotic use in livestock farming has not been considered a major public health problem in terms of emerging antibiotic-resistant strains. The lack of adequate data and thorough surveillance make the true impact of antibiotic use in livestock challenging to assess (Landers et al. 2012). Thus, multidrug antibiotic resistance in farming practices might be associated with antibiotic misuse and unsanitary farming practices, as has been reported in Monteria, Colombia (Máttar et al. 2009). In this study, *E. coli* and *S. aureus* were detected in raw milk; their presence was associated with poor milking practices and the cattle’s exposure to the indiscriminate use of veterinary drugs. Thus, the milking routine is a key determinant of quality milk production. Unsanitary handling of the cow’s udder during milking is a frequent source of contamination with *S. aureus*, which is recognized as a microbial contaminant (Kateete et al. 2013). GFP guidelines address those problems through the implementation of protocols through which livestock farmers can control environmental bacteria such as *E. coli* and *S. aureus*, which primarily tend to colonize the teat skin (Azevedo et al. 2016).

The emergence of antibiotic-resistant and multidrug-resistant bacterial contaminants in milk is further complicated by the sale of raw milk to local consumers or small-scale traders in the Sumapaz region. In other settings, the marketing of raw milk has been associated with major health problems, such as the emergence of *E. coli* O157:H7 strains (Addo et al. 2011; Mhone et al. 2011; Rasheed et al. 2014; Amagliani et al. 2016). Concerning antibiotic susceptibility, OXY, ERY, and AMX do not seem to play a significant selective role against microbial contamination in milk; rather, they contribute to increased bacterial resistance or create selective pressure for microorganisms that are already resistant to those drugs. Therefore, livestock farmers should be trained in sustainable livestock production, particularly with regard to antibiotic stewardship (Chang et al. 2015). Contaminated milk might lead to the emergence of strains with extended antibiotic resistance and the potential to spread among human and animal populations, which could influence the farm production environment (Haran et al. 2012; Iweriebor et al. 2015).

Screening for other food-borne pathogens, such as *Brucella abortus* and *Mycobacterium bovis—*the causative agents of brucellosis and bovine TB, respectively—was also performed in this study. Twenty-three cases of brucellosis and none of bovine TB were detected. Brucellosis is a zoonotic infection that is contagious to humans and requires an expensive and prolonged treatment regimen. In Colombia, brucellosis has been recognized as a considerable challenge faced by livestock farmers. The bovine brucellosis incidence in the Caribbean region has been estimated to range from 3.7 to 12.7% in animals and farms (Aznar et al. 2014). Approximately 4% of humans working in slaughterhouses in Tolima have been diagnosed with brucellosis (Morales and Combariza 2004). Additionally, the real prevalence of brucellosis might be underestimated in Colombia because of a lack of proper registration and a need for specific and sensitive diagnostic methods (Higgins et al. 2012). Second, no cases of bovine TB were detected, which seems to reflect the progress of the Bovine Tuberculosis Eradication Program in Colombia, which has maintained the bovine TB prevalence below 1% (De Waard 2010). However, because the CFT is a screening test, negative results cannot be considered absolute proof of the absence of bovine TB in a given cow or herd. Thus, CFT only provides an indication if an animal develops an immune response to the antigen for *M. bovis* (De Kantor and Ritacco 2006).

Epidemiological surveillance of livestock production might support the government’s planning initiatives and policies, programs, and procedures to protect animal and human health (Henrioud 2011; Donado-Godoy et al. 2015). The Ministry of Agriculture and Rural Development in Colombia assesses some economic benefits for livestock farmers who implement GFPs. Thus, GFP acceptance has been difficult to promote among small-holder farmers in the Sumapaz region because of the initial economic investment, the lack of knowledge about cost-benefits, the need for follow-up registration, and a lack of understanding about practices. Once livestock farmers accepted the advantages, it was possible to optimize the production and commercialization of dairy products and promote ecological practices (Seufert et al. 2012). Problems regarding the commercialization of contaminated dairy products and raw milk or infected cattle still persist in the Sumapaz region. Thus, we expect that the training and implementation of GFPs would contribute to the development of an agriculture sustainability model for livestock farmers in the Sumapaz region.

In conclusion, screening of food-borne and zoonotic pathogens is pivotal to control accurately potential outbreaks in the Sumapaz region. Although TB cases were not identified, brucellosis remains as a frequent zoonotic disease (4.2% seroprevalence). Occurrence of multidrug-resistant *E. coli* strains is relatively high and could represent a source of infection for farmers and consumers of dairy products. Further studies to identify the molecular basis of antibiotic resistance are required.

AMX, amoxicillin; CFTs, caudal-fold tuberculin tests; CIP, ciprofloxacin; DOX, doxycycline; EFX, enrofloxacin; ELISA, enzyme-linked immunosorbent assay; ERY, erythromycin; FOF, fosfomycin; GEN, gentamicin; GFPs, good farming practices; ICA, Colombian Agriculture and Livestock Institute; NCCLS, National Committee for Clinical Laboratory Standards; NOR, norfloxacin; OXY, oxytetracycline; PPD, purified protein derivative; SXT, trimethoprim-sulfamethoxazole; TB, tuberculosis
